# What can the Global South learn from Canada’s innovations in pain science?

**DOI:** 10.1080/24740527.2025.2541108

**Published:** 2025-09-25

**Authors:** Jose Eric M. Lacsa

**Affiliations:** Theology and Religious Education Department, De La Salle University, Manila, Philippines

**Keywords:** Pain research, Global South, Philippine healthcare, pain management innovation, Trainee-Led research

## Abstract

Canada’s advancements in pain research, characterized by innovative education, clinical care, and trainee-led scholarship, offer valuable insights for the Global South. This article examines key initiatives highlighted in a recent *Canadian Journal of Pain* special issue, including multidisciplinary approaches, patient-centered care, and the development of accessible pain assessment tools. By contextualizing these innovations within the Philippine healthcare landscape, the article explores challenges such as limited access, cultural perceptions of pain, and under-resourced pain management systems. Emphasizing the importance of narrative-driven and culturally sensitive methodologies, it advocates for integrating indigenous knowledge and community participation into pain research and care. Furthermore, the article underscores the critical role of nurturing early-career researchers and fostering cross-sector collaboration to build sustainable pain research ecosystems. Ultimately, this reflection invites Global South countries to adapt and co-create pain science innovations, contributing to a more inclusive and globally connected understanding of pain management. The article serves as a call to reimagine pain research that bridges local realities with global expertise, fostering equitable health outcomes across diverse populations.

## RÉSUMÉ

Les avancées du Canada dans la recherche sur la douleur, portées par une formation novatrice, des soins cliniques et des initiatives de recherche dirigées par des stagiaires, offrent des enseignements précieux pour les pays du Sud. Cet article examine les principales initiatives mises en lumière dans un récent numéro spécial de la *Revue canadienne de la douleur*, notamment les approches multidisciplinaires, les soins centrés sur le patient et la mise au point d'outils d'évaluation de la douleur accessibles. En contextualisant ces innovations dans le système de santé philippin, il met en évidence des défis tels que l'accès limité, les perceptions culturelles de la douleur et le sous-financement des systèmes de prise en charge. Soulignant l'importance de méthodologies narratives et sensibles à la culture, il plaide pour une meilleure intégration des savoirs autochtones et de la participation communautaire dans la recherche et les soins. L'article souligne également le rôle essentiel du soutien aux chercheurs en début de carrière et de la collaboration intersectorielle pour bâtir des écosystèmes de recherche sur la douleur. Enfin, il invite les pays du Sud à adapter et co-créer des innovations en science de la douleur, contribuant ainsi à une approche plus inclusive et globale de la prise en charge de la douleur à l’échelle mondiale. Cette réflexion constitue un appel à réinventer la recherche sur la douleur en reliant les réalités locales au savoir scientifique mondial, afin de favoriser des résultats de santé équitables pour des populations diverses.

To the Editor

I would like to offer an observation that may enrich the ongoing discourse in pain research, particularly in response to the recent article by Hance Clarke et al., *“The Future Is Bright: Highlighting Trainee Contributions to the Canadian Journal of Pain.”*^1^ The article asserts that Canada has established itself as a global leader in pain research and continues to promote innovation, excellence, and leadership in pain education, clinical care, and research through initiatives like this special issue featuring trainee-led studies across diverse populations and methodologies. These achievements not only reflect the strength of Canada’s scientific ecosystem but also pose a generative challenge: how might other nations, particularly in the Global South, draw from and reimagine these insights in their own contexts?

In the Philippines, pain is a deeply layered experience, shaped by disparities in healthcare access, cultural attitudes toward suffering, and the under-resourcing of pain management infrastructure.^2^ The complexity of fibromyalgia, for instance, echoes findings by De Clifford-Faugère et al. regarding gaps between clinical guidelines and patient treatment. Locally, misdiagnoses, stigma, and inadequate follow-up care remain barriers to effective pain relief, particularly for women and low-income populations.

Narrative-centered approaches explored by Canadian scholars, such as Nishikawara et al.’s examination of patient invalidation and Serota et al.’s ethics of care surrounding Medical Assistance in Dying (MAiD), resonate strongly in our setting, where the emotional and spiritual dimensions of pain are often entwined.^3^ Their work underscores the importance of listening to patients not only as subjects of care, but as partners in shaping it. In the Philippine context, this could mean integrating folk medicine and faith-based practices with clinical care, respecting the lived realities of patients often excluded from formal health discourse.

The development of caregiver-friendly tools such as the CPOT-Fam also holds potential for adaptation in low-resource settings. Filipino families, who often serve as primary caregivers due to systemic health gaps, could benefit greatly from similar innovations. Localized and culturally sensitive tools can empower non-clinicians to participate more confidently in pain assessment and care.^4^

Equally important is Canada’s investment in young researchers. By mentoring trainees and fostering cross-disciplinary collaborations, they are laying foundations for long-term systemic impact.^5^ The Philippines can take a cue from this by strengthening support for early-career researchers in pain science, encouraging participatory, community-based projects that amplify marginalized voices.

Canada’s leadership in pain research is not a distant ideal but an invitation to rethink our own possibilities. By drawing inspiration while remaining grounded in local contexts, countries like the Philippines can contribute meaningfully to global pain scholarship – building bridges that span both knowledge and compassion.
